# Supplementary material for: Deletion of wfs1 Impairs Oligodendrocyte Precursor Cells Dorsal Distribution and Myelination Through the wfs1-hmgcs1 Axis in Zebrafish
Source: Biology (Basel). 2026 Aug 16;15(16):1402. doi: 10.3390/biology15161402 (PMC13510151; doi:10.3390/biology15161402)
Supplement: Supplementary file 1 [file biology-15-01402-s001.zip › File S1-Transcriptome sequencing data and differentially expressed genes (DEGs) in wfs1-- zebrafish at 5 dpf/2.KEGG/wfs1KOvsWT/ALL/src/dre03020.html]

dre03020


- dre:325603

- Down regulated genes

325603(-2.47138728794198)

- dre:562329

- Up regulated genes

562329(1.23033679822858)

- dre:394162

- Up regulated genes

394162\_1(1.13013950134542)

- Down regulated genes

394162(-2.30704898724063)

Close
